# Colossal Magnetic‐Field‐Induced Anomalous Hall Conductivity in a Kagome Antiferromagnet YMn_6_Sn_6_

**DOI:** 10.1002/advs.77738

**Published:** 2026-09-21

**Authors:** Minhyuk Choi, Hoil Kim, Minki Sung, Hyeongwoo Seo, Joonyoung Choi, Jaeyoung Kim, Younjung Jo, Han Woong Yeom, Kyung‐Hwan Jin, Jun Sung Kim

**Affiliations:** ^1^ Department of Physics Pohang University of Science and Technology Pohang Republic of Korea; ^2^ Center for Artificial Low Dimensional Electronic Systems Institute for Basic Science (IBS) Pohang Republic of Korea; ^3^ Institute for Materials Research Tohoku University Sendai Japan; ^4^ Department of Physics Kyungpook National University Daegu Republic of Korea; ^5^ Department of Physics and Research Institute of Materials and Energy Sciences Jeonbuk National University Jeonju Republic of Korea

**Keywords:** antiferromagnetism, berry connection and curvature, condensed matter physics, dirac cone, electronic band structure, electronic properties, flat band, frustration, magnetic field, spintronics

## Abstract

Kagome‐lattice magnets provide a fertile ground for exploring exotic electronic phases due to topological band structures and complex magnetic orders. Yet, how these ingredients dictate unconventional magnetotransport responses, crucial for possible spintronic applications, has remained elusive. Here, we show that a prototypical kagome antiferromagnet ${\mathrm{YMn}}_{6}{\mathrm{Sn}}_{6}$ exhibits significant magnetic‐field‐driven redistribution of Berry curvature and the colossal anomalous Hall conductivity (AHC). Under high magnetic fields, spin canting induces a pronounced spin splitting of the flat and Dirac bands near the Fermi level, dramatically enhancing the Berry curvature. The resulting AHC reaches ∼ 2.5 × 10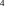 S/cm, far exceeding the intrinsic Berry curvature predictions and ranking among the highest reported in magnetic systems. This colossal AHC enhancement is attributed to a synergy between field‐tuned Berry curvature and enhanced skew scattering in the clean limit. Our findings highlight the critical role of magnetic‐field‐controlled spin splitting in engineering Berry curvature and anomalous magnetotransport in clean kagome magnets.

## Graphical abstract

Magnetic‐field‐driven spin canting in the kagome antiferromagnet ${\mathrm{YMn}}_{6}{\mathrm{Sn}}_{6}$ reshapes flat and Dirac bands near the Fermi level, redistributing Berry curvature and producing colossal anomalous Hall conductivity. The study reveals how magnetic configuration and clean‐limit skew scattering cooperate to amplify Hall transport, offering a route to tune topological responses in kagome magnets.

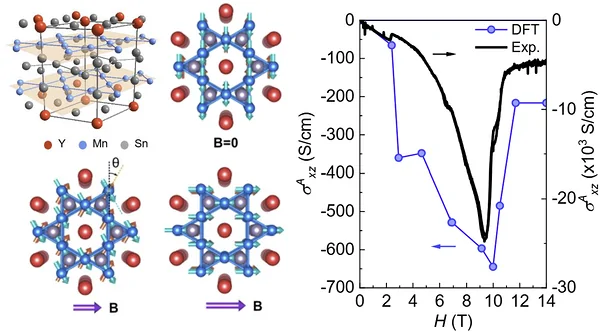

## 1 Introduction

Kagome‐lattice magnets have recently attracted extensive attention due to their potential to host exotic magnetotransport phenomena [1, 2]. The kagome lattice, composed of corner‐sharing triangles, inherently supports distinct electronic structures including Dirac cones [3, 4, 5, 6, 7, 8, 9], flat bands [7, 8, 10, 11, 12, 13], and van Hove singularities [4, 5, 6, 14, 15], providing nontrivial topological states and substantial Berry curvature effects. Furthermore, strong geometrical magnetic frustration, intrinsic to kagome lattices, often stabilizes nontrivial magnetic orders, such as noncollinear antiferromagnetism [16, 17, 18, 19, 20, 21, 22, 23, 24, 25, 26, 27, 28], spin liquids [29, 30], and skyrmion lattices [31, 32]. Consequently, these topological electronic features, coupled with complex spin textures, can lead to anomalous transport properties such as large anomalous Hall effects (AHE) [3, 19, 20, 33, 34, 35, 36] and topological Hall effects (THE) [21, 22, 37, 38], making kagome magnets promising candidates for next‐generation spintronic devices. However, understanding the underlying mechanism of their rich magnetotransport behaviors has remained elusive.

Among recently explored kagome magnets, ${\mathrm{YMn}}_{6}{\mathrm{Sn}}_{6}$ has emerged as a prototypical kagome antiferromagnet hosting complex magnetic structures, including an incommensurate spiral magnetic order and magnetic‐field‐induced spin canting, which significantly influence its magnetotransport properties [21, 22, 23, 24, 25, 26, 27, 28, 39]. Previous studies reported a large AHE with strong variation with external magnetic field, which has been attributed to the THE, explained by magnon‐induced spin scalar chirality at high temperatures [21]. However, first‐principles calculations have predicted the presence of a Dirac band near the Fermi level in ${\mathrm{YMn}}_{6}{\mathrm{Sn}}_{6}$, subsequently confirmed via angle‐resolved photoemission spectroscopy (ARPES) [23]. This Dirac band originating from the kagome lattice implies a possible intrinsic Berry curvature contribution to the observed AHE, but such a possibility has been largely overlooked. Additionally, due to its high electrical conductivity at low temperatures, extrinsic contributions like skew scattering cannot be neglected [40, 41, 42]. Consequently, the origin behind the pronounced AHE and its strong magnetic field dependence are unclear, calling for further experimental and theoretical investigations.

Here, we combine detailed magnetotransport measurements, ARPES, and density functional theory (DFT) calculations to investigate the magnetic‐field‐induced modulation of momentum (k)‐space Berry curvature and anomalous Hall conductivity (AHC) in ${\mathrm{YMn}}_{6}{\mathrm{Sn}}_{6}$. We found that strong variation of Berry curvature is induced by systematic changes of the spin‐polarized Dirac bands near the Fermi level due to spin canting. The dramatic enhancement of the AHC of ${\mathrm{YMn}}_{6}{\mathrm{Sn}}_{6}$ both above and below magnetic saturation fields at low temperatures is attributed to this Berry curvature modulation, amplified by skew scattering in the clean limit. The resulting record high AHC of ${\mathrm{YMn}}_{6}{\mathrm{Sn}}_{6}$ demonstrates that the interplay of k‐space Berry curvature and the associated skew scattering offers a promising route to enhancing the magnetotransport responses in kagome magnets.

## 2 Electronic and Magnetic Properties

**FIGURE 1:**
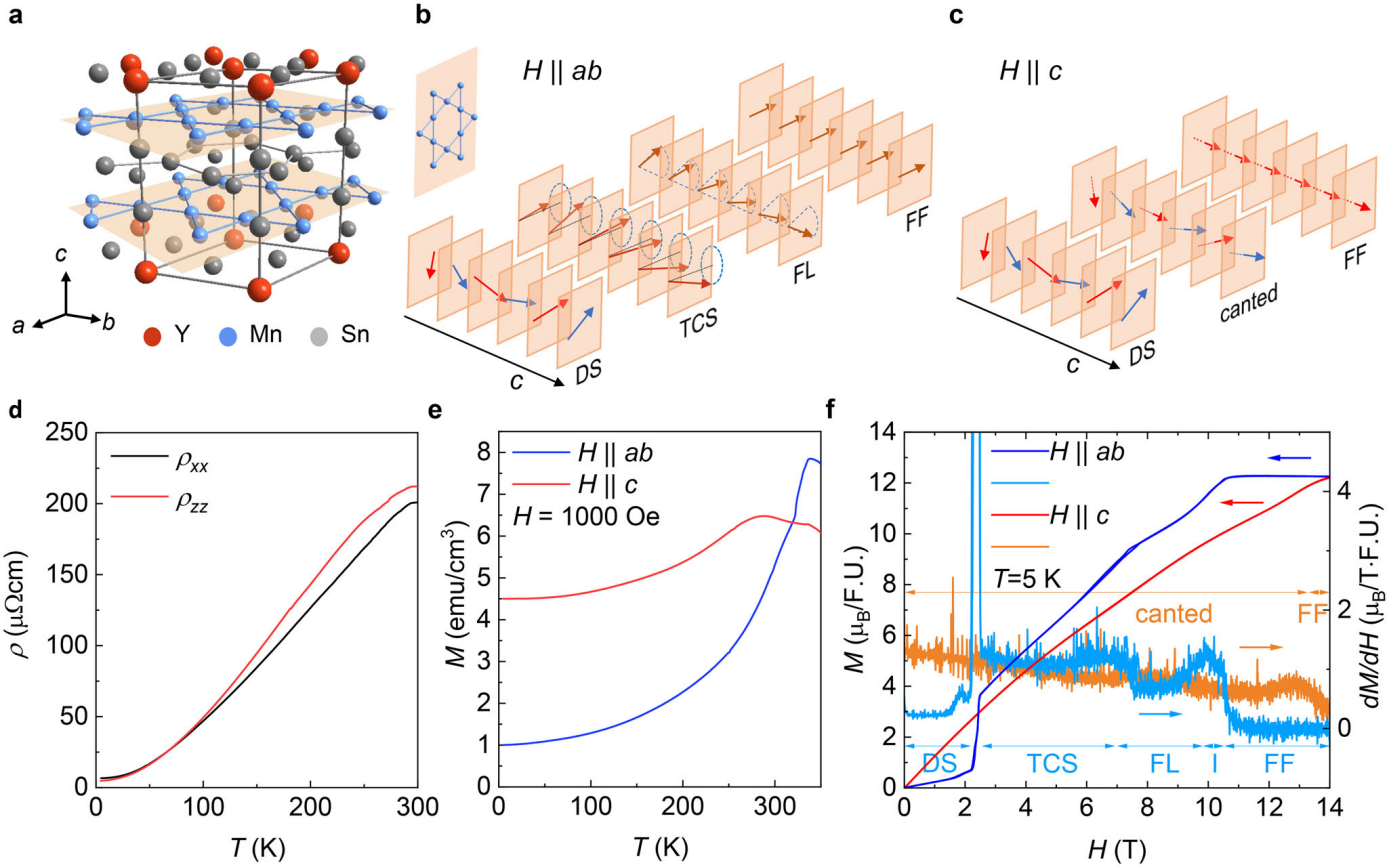
Crystal structure, field‐dependent magnetic structures, and transport properties of ${\mathrm{YMn}}_{6}{\mathrm{Sn}}_{6}$. (a) Crystal structure of ${\mathrm{YMn}}_{6}{\mathrm{Sn}}_{6}$, showing the kagome layers (shadow plane) composed of Mn atoms. Mn kagome layers are sandwiched with Sn and Y‐Sn layers ordered as ${[\mathrm{Mn}}_{3}{\mathrm{Sn}][\mathrm{Sn}}_{3}{][\mathrm{Mn}}_{3}{\mathrm{Sn}][\mathrm{Sn}}_{2}Y]$. (b) Schematic representations of magnetic spin structures for H∥ab, showing the field‐induced evolution from the distorted spiral (DS) to transverse conical spiral (TCS), fan‐like (FL), and forced ferromagnetic (FF) phases. (c) Schematic representations of the magnetic spin structures for H∥c, showing the continuous spin canting from the DS state toward the FF state. (d) Temperature dependence of electrical resistivity measured along the ab‐plane ($\rho_{xx}$) and along the c‐axis ($\rho_{zz}$). (e) Temperature‐dependent magnetic susceptibility measured under magnetic fields of 1000 Oe, applied along the ab‐plane and the c‐axis. The Néel temperature ($T_{N}$) is identified to be approximately 345 K for both field directions. (f) Magnetic field dependence of magnetization at 5 K for magnetic fields applied along the ab‐plane and the c‐axis. For H∥ab, the derivative dM/dH is also shown to identify the magnetic phase boundaries between the DS, TCS, FL, I (intermediate), and FF phases, whereas M(H) for H∥c exhibits a monotonic evolution toward the FF state.

${\mathrm{YMn}}_{6}{\mathrm{Sn}}_{6}$ crystallizes into a hexagonal kagome structure with space group P6/mmm, characterized by alternating layered arrangements as depicted in Figure 1a. Mn atoms form distinct kagome planes, sandwiched by Sn‐only and mixed Y‐Sn layers, in an alternating stacking sequence. This structural arrangement is critical for stabilizing various magnetic orders with competing exchange interactions, reflected in the complex field‐dependent magnetic structures (Figure 1b). Magnetization measurements on our single crystals reveal an antiferromagnetic phase transition at Néel temperature $T_{N}$ ≈ 345 K and also significant anisotropy between the in‐plane (ab‐plane) and out‐of‐plane (c‐axis) directions (Figure 1e,f), in good agreement with the previous reports [21, 23, 26, 27, 28, 39]. Temperature‐dependent electrical resistivity along the ab‐plane and c‐axis, denoted as $\rho_{xx}$ and $\rho_{zz}$, respectively, exhibit clear metallic behaviors (Figure 1d). The residual resistivity ratio ρ(300 K)/ρ(5 K) reaches to ∼ 44 for $\rho_{zz}$ and ∼ 30 for $\rho_{xx}$. These results are also comparable with those reported in the previous experimental reports [21, 22, 23, 24, 25, 28]. The resulting conductivity at low temperatures reaches $∼2.1\times 10^{5}$ S/cm, and $∼1.5\times 10^{5}$ S/cm, for $\rho_{zz}$ and $\rho_{xx}$, respectively, which are similar to those of many ferromagnets close to the clean regime [42]. These observations indicated that in a high quality single crystal of ${\mathrm{YMn}}_{6}{\mathrm{Sn}}_{6}$, extrinsic scattering contribution, including skew scattering, can be significant for the observed AHE, discussed in detail below.

The low temperature phase of ${\mathrm{YMn}}_{6}{\mathrm{Sn}}_{6}$ exhibits an incommensurate magnetic order with a distorted spiral structure. Under in‐plane magnetic fields, this distorted spiral (DS) changes to the transverse conical spiral (TCS) phases. Upon further increasing the magnetic field, transitions into fan‐like (FL), and intermediate (I) phases, accompanied by distinct spin configurations as illustrated schematically in Figure 1b. Eventually, forced ferromagnetic (FF) phases are observed above the magnetization saturation field. Our magnetization measurements M(H) taken at 5 K for H ∥ ab show four distinct anomalies at H ≈ 2.5, 7.0, 9.9, and 10.5 T, which are more clearly visible in the field‐derivative curve dM(H)/dH and is consistent with the previously established magnetic phase diagram [21, 22, 24, 25, 26, 27, 28, 39]. For H ∥ c, in contrast, spin canting in the DS phase results in monotonic field dependence of M(H) below the saturation field at ∼ 13.5 T. Excellent agreement with the previous reports confirms the good quality of our crystals [21, 22, 24, 25, 27, 28].

## 3 Electronic Structure Without Magnetic Fields

**FIGURE 2:**
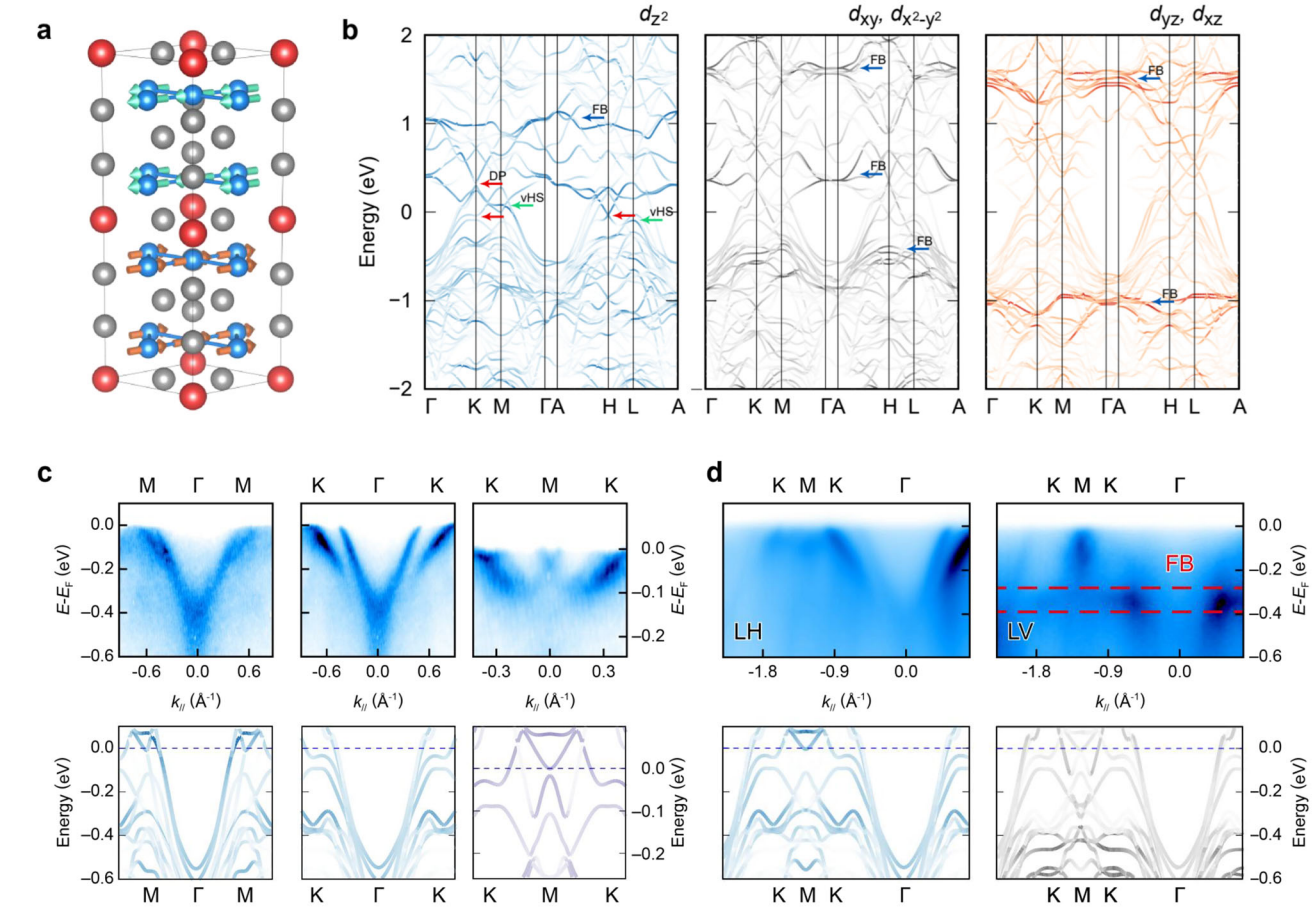
Electronic structure of ${\mathrm{YMn}}_{6}{\mathrm{Sn}}_{6}$: kagome‐derived bands and ARPES spectra. (a) Crystal structure of ${\mathrm{YMn}}_{6}{\mathrm{Sn}}_{6}$ used for the DFT calculations, modeled with a double‐layer AFM spin configuration. Mn moments are ferromagnetically aligned within each kagome bilayer but antiferromagnetically coupled between adjacent bilayers along the c‐axis. To represent this ordering, the unit cell is doubled along the z direction. (b) Orbital‐resolved band structures calculated with spin‐orbit coupling for the double‐layer AFM model: Mn $d_{z^{2}}$ (left, blue), Mn $d_{xy}$, $d_{x^{2}-y^{2}}$ (middle, gray), and Mn $d_{yz}$, $d_{xz}$ (right, orange) characters. Red, blue, and green arrows indicate representative kagome‐derived features, namely the Dirac points (DP), flat bands (FB), and van Hove singularities (vHS), respectively. (c) ARPES intensity along high‐symmetry directions measured at hν=80 eV and T=10 K on the kagome‐terminated surface, compared with DFT calculations including spin‐orbit coupling considering a double‐layer AFM model. The measured dispersion is well captured by the calculations, including Dirac‐like features near the K points. (d) Polarization‐dependent ARPES intensity maps measured along high‐symmetry ΓK direction at hν=138 eV and T=70 K. Flat band at a binding energy of ∼ 0.4 eV is prominently observed under LV polarization, reflecting its orbital selectivity. Orbital‐resolved DFT calculations show that flat band exhibits strong $d_{xy}$ and $d_{x^{2}-y^{2}}$ contributions.

To capture the essential low‐energy physics of ${\mathrm{YMn}}_{6}{\mathrm{Sn}}_{6}$ while maintaining computational feasibility, we modeled the experimentally reported incommensurate “distorted spiral” magnetic structure by a commensurate double‐layer antiferromagnetic (AFM) configuration (Figure 2a). In this model, Mn spins within each kagome bilayer are ferromagnetically aligned, while successive bilayers are coupled antiferromagnetically along the c‐axis, requiring a doubling of the primitive unit cell in the z‐direction. This approximation preserves the key interlayer exchange symmetry breaking while avoiding the computational complexity of fully incommensurate spin textures. The resulting spin‐orbit‐coupled band structure (Figure 2b) exhibits characteristic kagome‐derived electronic features, including Dirac‐like dispersions, nearly flat bands, and saddle point features. Importantly, these kagome‐derived bands exhibit distinct orbital characters. The Dirac‐like bands closest to the Fermi level near the K point are predominantly derived from Mn $d_{z^{2}}$ orbital. In contrast, the flat band observed around E≈−0.4 eV is dominated by the in‐plane Mn $d_{xy}$ and $d_{x^{2}-y^{2}}$ orbitals, while additional flat‐band features farther from the Fermi level contain substantial $d_{yz}$ and $d_{xz}$ contributions. This orbital‐selective character reflects the multi‐orbital nature of the kagome electronic structure in ${\mathrm{YMn}}_{6}{\mathrm{Sn}}_{6}$.

To verify the commensurate double‐layer AFM configuration, we measured ARPES spectra and compared them with the corresponding DFT calculations. Figure 2c shows the ARPES spectra measured along high‐symmetry directions on the kagome‐terminated surface, which exhibit excellent agreement with DFT calculations incorporating the double‐layer AFM ordering, including the overall band dispersion and Dirac‐like dispersions near the K points. We further identify a flat band with a binding energy of 0.3– 0.4 eV (Figure 2d), another electronic fingerprint of the kagome lattice. The flat band exhibits strong dependence on the polarization of incident light, appearing only in the linear vertical (LV) polarization, which selectively excites in‐plane orbitals (Figure S6). Consistently, DFT calculations show that the flat band near −0.4 eV, close to the Fermi level, is dominantly derived from the $d_{xy}$ and $d_{x^{2}-y^{2}}$ orbitals. These agreements indicate that the commensurate double‐layer AFM model reliably captures essential features of the experimentally observed electronic structures in ${\mathrm{YMn}}_{6}{\mathrm{Sn}}_{6}$. Additional spectral features in the experiments are attributed to Sn surface states, as identified in the Fermi surface and Sn 4d core‐level spectra (Figure S5). Distinct electronic structures can arise depending on the cleavage surface were noticed previously [5, 6, 43, 44].

## 4 Electronic Structure Modulation by Field‐Induced Spin Canting

**FIGURE 3:**
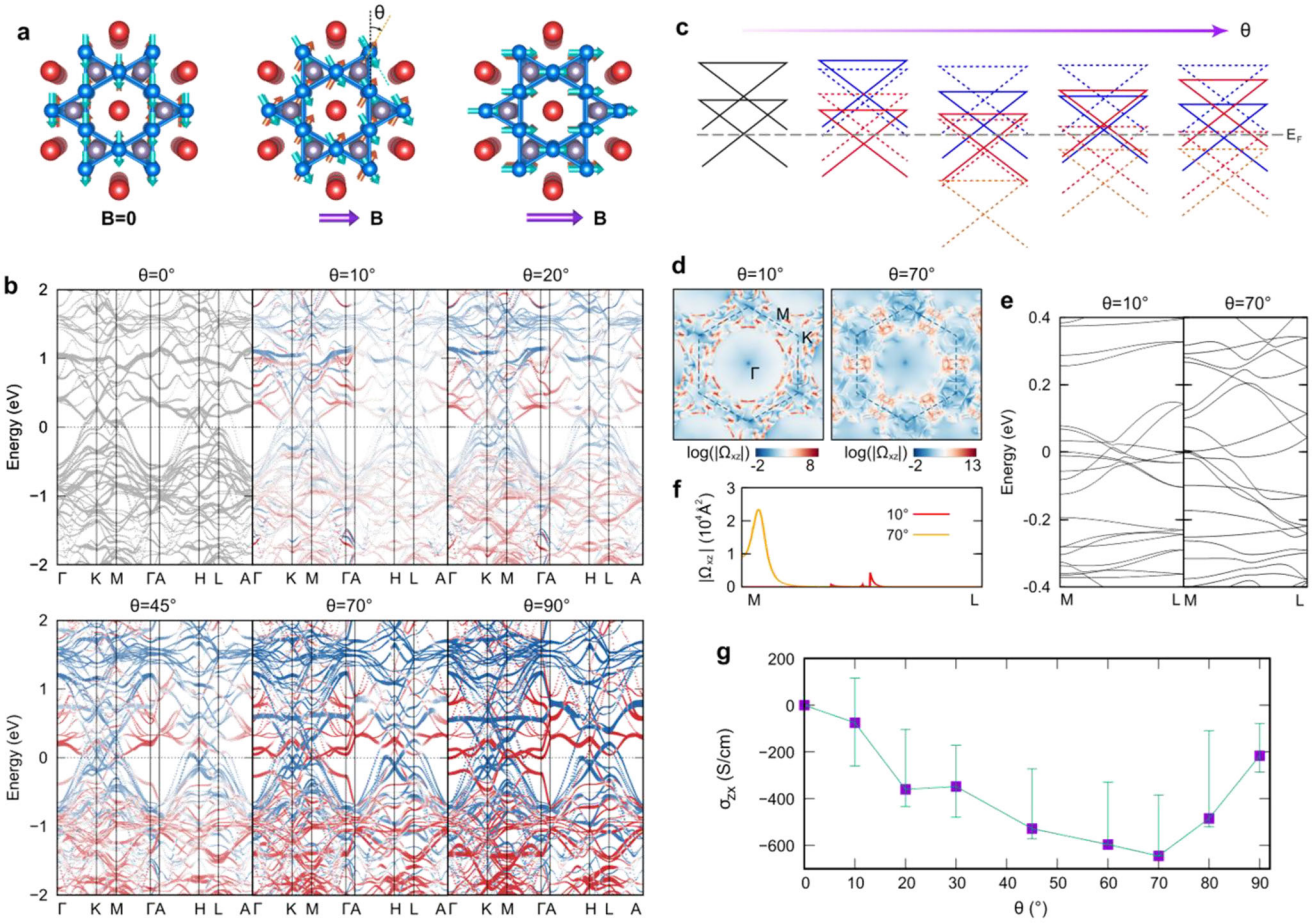
Evolution of kagome bands, Berry curvature, and quantum AHC under spin canting in ${\mathrm{YMn}}_{6}{\mathrm{Sn}}_{6}$. (a) Schematic illustration of the double‐layer antiferromagnetic configuration under an in‐plane magnetic field. (b) θ‐dependent band structures projected onto the Mn $d_{z^{2}}$ orbital (dot size proportional to orbital weight). The red‐blue color gradient indicates spin polarization along the field direction. (c) Schematic representation of the θ‐driven evolution of the kagome band manifold, showing the progressive spin splitting and downward shift of the flat bands. (d) Log scale map of Berry curvature distribution $|\Omega_{xz}\left|=\right|\sqrt{\Omega_{x}^{2}+\Omega_{z}^{2}}$ in the $k_{z}$=0 plane for $\theta =10^{\circ }$ and $\theta =70^{\circ }$, respectively. At $\theta =70^{\circ }$, large Berry curvature hotspots emerge near the M point. (e) Band structures along the M‐L path for $\theta =10^{\circ }$ and $\theta =70^{\circ }$. A sizable SOC‐induced gap opens near the M point at $\theta \approx 70^{\circ }$, correlating with the Berry curvature enhancement. (f) Berry curvature magnitude along the M‐L path for $\theta =10^{\circ }$ and $\theta =70^{\circ }$, showing a strong peak near M at $\theta =70^{\circ }$. (g) Calculated quantum AHC, $\sigma_{xz}$, as a function of θ. Error bars reflect variations obtained by shifting the Fermi level by ±50 meV.

Having established the electronic structures at zero magnetic field, we now focus on the effect of an in‐plane magnetic field on the double‐layer AFM ground state. Compared to the response to an out‐of‐plane magnetic field, the more complex evolution of the magnetic structure under an in‐plane field (Figure 1b) is accompanied by a pronounced nonmonotonic field dependence of the Hall response, as discussed below, thereby providing a more direct basis for comparison between theory and experiment. To model it, we introduce a uniform spin canting angle θ, defined as the rotation of Mn spins from their zero‐field AFM orientation toward the field direction (Figure 3a and Figure S1). This approach captures the gradual transformation from AFM ($\theta =0^{\circ }$) to FM ordering ($\theta =90^{\circ }$) with increasing field strength. Two effects are expected from this spin canting on the electronic structure. First, systematic increase of spin splitting by Zeeman effect as net magnetic moment increases by spin canting. The other is band folding by unit cell doubling along the $k_{z}$ direction in double‐bilayer AFM configuration (Figure 2a) weakens and eventually disappears in the FF phase with a single bilayer in the unit cell. The calculated Mn $d_{z^{2}}$‐projected band structures (Figure 3b and Figure S2) indeed reveal that the kagome‐derived flat bands undergo progressive spin splitting and approach the Fermi level as θ increases. This shift is accompanied by enhanced dispersion along $k_{z}$, reflecting the evolution toward interlayer FM alignment. The schematic in Figure 3c summarizes these θ‐dependent band changes, highlighting the downward movement and spin splitting of the flat bands.

The systematic variation of the low energy structures with spin canting leads to significant modulation of Berry curvature. As shown in Figure 3d, we calculated the Berry curvature distribution $|\Omega_{xz}|$ in the $k_{z}=0$ plane. For $\theta =70^{\circ }$, large Berry curvature hotspots appear near the M point (Figure 3d and Figure S3), in stark contrast to the relatively weak distribution at $\theta_{M}=10^{\circ }$. This enhancement is linked to a pronounced spin‐orbit coupling (SOC)‐induced gap along the M–L direction at the Fermi level for $\theta_{M}\approx 70^{\circ }$ (Figure 3e), as confirmed by the sharp peak in Berry curvature along M‐L (Figure 3f and Figure S4). The calculated quantum AHC $\sigma_{zx}$ exhibits a strong $\theta_{M}$ dependence (Figure 3g), peaking near $\theta_{M}\approx 70^{\circ }$ in line with the Berry curvature maximum. This trend persists even when the Fermi level is shifted by ±50 meV, indicating robustness against moderate carrier doping. These results suggest that field‐induced spin canting in ${\mathrm{YMn}}_{6}{\mathrm{Sn}}_{6}$ can tune Berry curvature in the momentum space through controlled modification of the kagome band topology. Thus the field‐induced magnetic phase transitions, associated with jumps in the M(H) (Figure 1f) and spin canting angle $\theta_{M}$ result in the significant changes in the k‐space Berry curvature.

## 5 Colossal Anomalous Hall Conductivity

**FIGURE 4:**
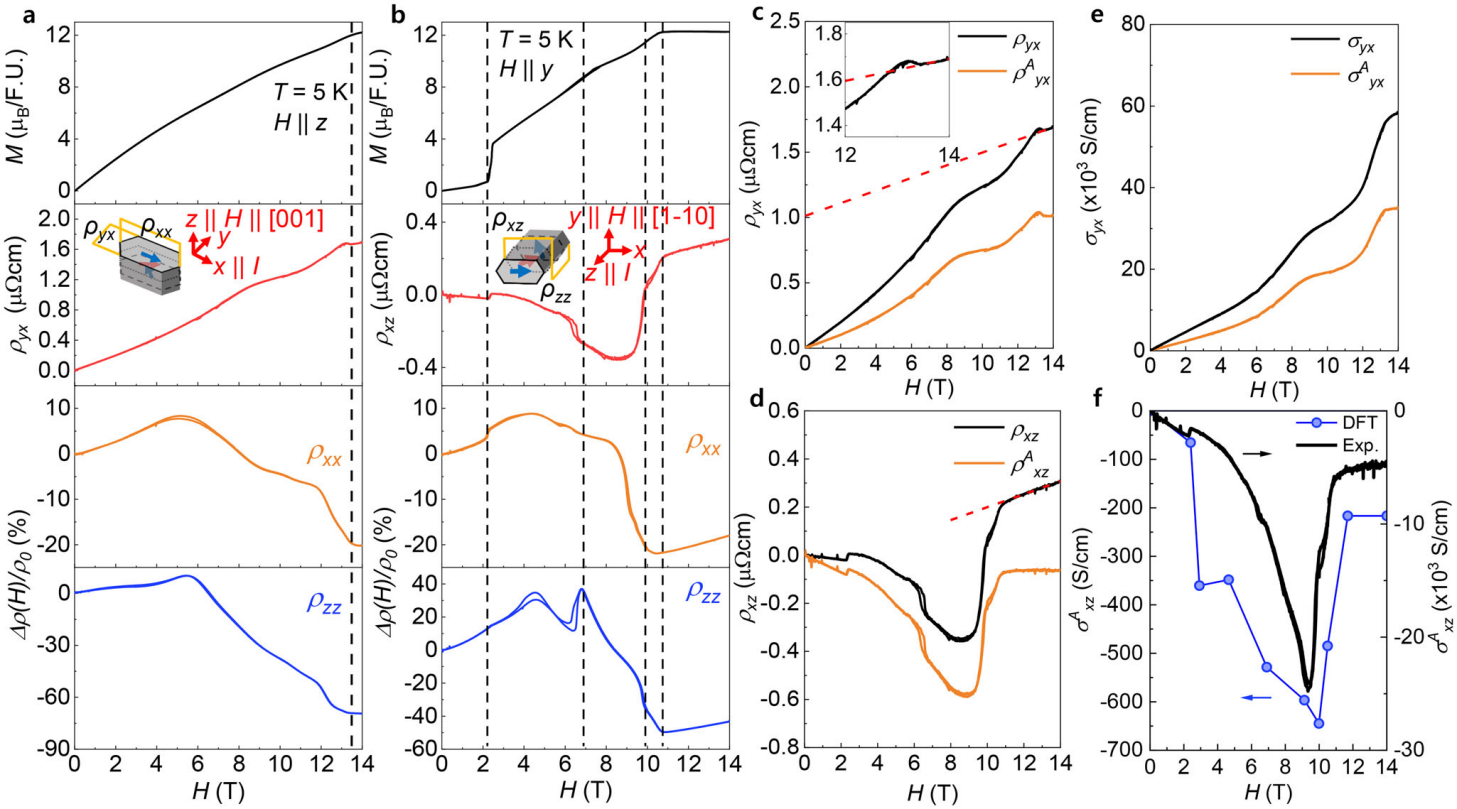
Magnetic‐field‐dependent anomalous Hall effect in ${\mathrm{YMn}}_{6}{\mathrm{Sn}}_{6}$. (a,b) Field dependence of magnetization(M), Hall resistivity ($\rho_{yx}$, $\rho_{xz}$) and longitudinal magnetoresistance ($\Delta \rho_{xx}/\rho_{xx}\left(0\right)$, $\Delta \rho_{zz}/\rho_{zz}\left(0\right)$) at T=5 K for two transverse Hall geometries with H∥z∥[001] (a) and H∥y∥[1−10] (b). For H∥z∥[001], $\rho_{\mathrm{yx}}$ is measured with current along the x direction, while $\Delta \rho_{xx}/\rho_{xx}\left(0\right)$ and $\Delta \rho_{zz}/\rho_{zz}\left(0\right)$ are tracked under the same field orientation. Slope changes in the transport curves near H≃13.5 T coincide with the saturation of M(H). For H∥y∥[1−10], $\rho_{xz}$ is measured with current along the z direction. The vertical dashed lines mark phase boundaries inferred from M(H) and the corresponding anomalies in $\rho_{xz}$, $\Delta \rho_{xx}/\rho_{xx}\left(0\right)$, and $\Delta \rho_{zz}/\rho_{zz}\left(0\right)$. Insets in a and b define the measurement geometries for the $\rho_{yx}$ and $\rho_{xz}$ Hall responses, respectively. (c,d) Hall resistivities, $\rho_{yx}$ and $\rho_{xz}$ (black) and the corresponding anomalous Hall resistivities, $\rho_{yx}^{A}$ and $\rho_{xz}^{A}$ (orange) for H∥z∥[001] (c) and H∥y∥[1−10] (d). The anomalous components are obtained by subtracting the normal Hall term $R_{0}H$, where $R_{0}$ is determined from linear fits to the high‐field magnetically saturated regime, indicated by red dashed lines. (e) Total Hall conductivity $\sigma_{yx}$ and anomalous Hall conductivity $\sigma_{yx}^{A}$ derived from the $\rho_{yx}$ geometry. (f) Field dependence of the anomalous Hall conductivity $\sigma_{xz}^{A}$ obtained from experiments (black), compared with the intrinsic Berry‐curvature contribution from DFT calculations (blue circles). Both curves show the same sign and a maximum magnitude near ∼9.5 T, indicating that spin‐canting‐induced Berry‐curvature redistribution captures the main field‐dependent trend, although the experimental magnitude substantially exceeds the intrinsic contribution.

To address the strong field‐dependent Berry curvature effect, we investigated the magnetotransport properties under different magnetic field orientations. Insets in Figures 4a, b illustrate schematic diagrams of two measurement configurations used for the magnetotransport experiments: one for the longitudinal ($\rho_{xx}$) and transverse ($\rho_{yx}$) resistivities with magnetic fields (H∥z∥[001]) and current (I∥x∥[100]) applied along the z‐ and x‐axes, respectively, and the other for the longitudinal ($\rho_{zz}$) and transverse ($\rho_{xz}$) resistivities with H∥y and I∥z. Clear anisotropic and nonmonotonic magnetotransport responses are observed under magnetic fields. For H∥z, while the M(H) data shows a smooth increase with magnetic field, clear changes in the slope of the magnetic‐field‐dependent resistivities ($\rho_{xx}\left(H\right)$ and $\rho_{yx}\left(H\right)$) are observed. This observation is consistent with significant variations in the electronic structure at the Fermi level due to spin canting. For H∥y, even more complex magnetic field dependencies of $\rho_{zz}$ and $\rho_{xz}$ are observed, showing step‐like features associated with field‐induced magnetic phase transitions. These results strongly suggest that magnetotransport properties are sensitive to both electronic structure variations caused by spin canting and the underlying magnetic structures.

The Berry curvature effect on magnetotransport can be monitored by AHC as shown in Figure 4e,f. To quantify the AHC, we first separate the ordinary Hall component, which arises from the Lorentz force‐driven charge deflection, from the measured transverse resistivities ($\rho_{\mathrm{yx}}$ and $\rho_{xz}$). For both configurations, the Hall resistivity can be described by $\rho_{H}=\rho_{N}+\rho_{A}=R_{H}B+\rho_{A}$ [19, 35, 36, 45, 46, 47, 48, 49], where $R_{H}$ is the Hall coefficient and $\rho_{A}$ is the anomalous contribution. The value of $R_{H}$ was obtained by fitting the data in the high‐field regions, H>13.5 T for H∥z and H>10.7 T for H∥y, where the magnetization is saturated, allowing $\rho_{A}$ to be treated as a field‐independent constant. These fittings yield $R_{H}$ ∼ 4.9 × 10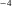 ${\mathrm{cm}}^{3}/C$ for H∥z and $R_{H}$ ∼ 2.6 × 10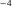 ${\mathrm{cm}}^{3}/C$ for H∥y, typical values of metals with large Fermi surfaces. We note that while the limited field range above $H_{\mathrm{sat}}$ for H∥z may introduce minor uncertainty in $R_{H}$, the obtained $R_{H}$ value for H∥y is reliable due to a sufficiently wide fitting range. Subsequently, the AHC, $\sigma_{ij}^{A}$, is calculated by subtracting the normal contribution $\sigma_{ij}^{N}=R_{H}B/\rho^{2}$ from the total Hall conductivity $\sigma_{ij}$, using the relationship $\sigma_{ij}^{A}\approx -\rho_{ij}^{A}/\left(\rho_{ii}\rho_{jj}\right)$, where i and j represent respective crystallographic axes. The resulting anomalous component $\sigma_{xz}^{A}$, illustrated as the black line in Figure 4f, exhibits significant variation with magnetic field, reaching a maximum value of $∼2.5\times 10^{4}$ S/cm in the FL phase. These values are much larger than typical AHC values found in various magnetic materials [40, 41, 50]. The pronounced enhancement, distinct anisotropy, and field‐dependent evolution of the AHC clearly highlight the critical roles of field‐induced spin canting, Berry curvature redistribution, and nontrivial magnetization structures in governing the magnetotransport properties of ${\mathrm{YMn}}_{6}{\mathrm{Sn}}_{6}$.

**FIGURE 5:**
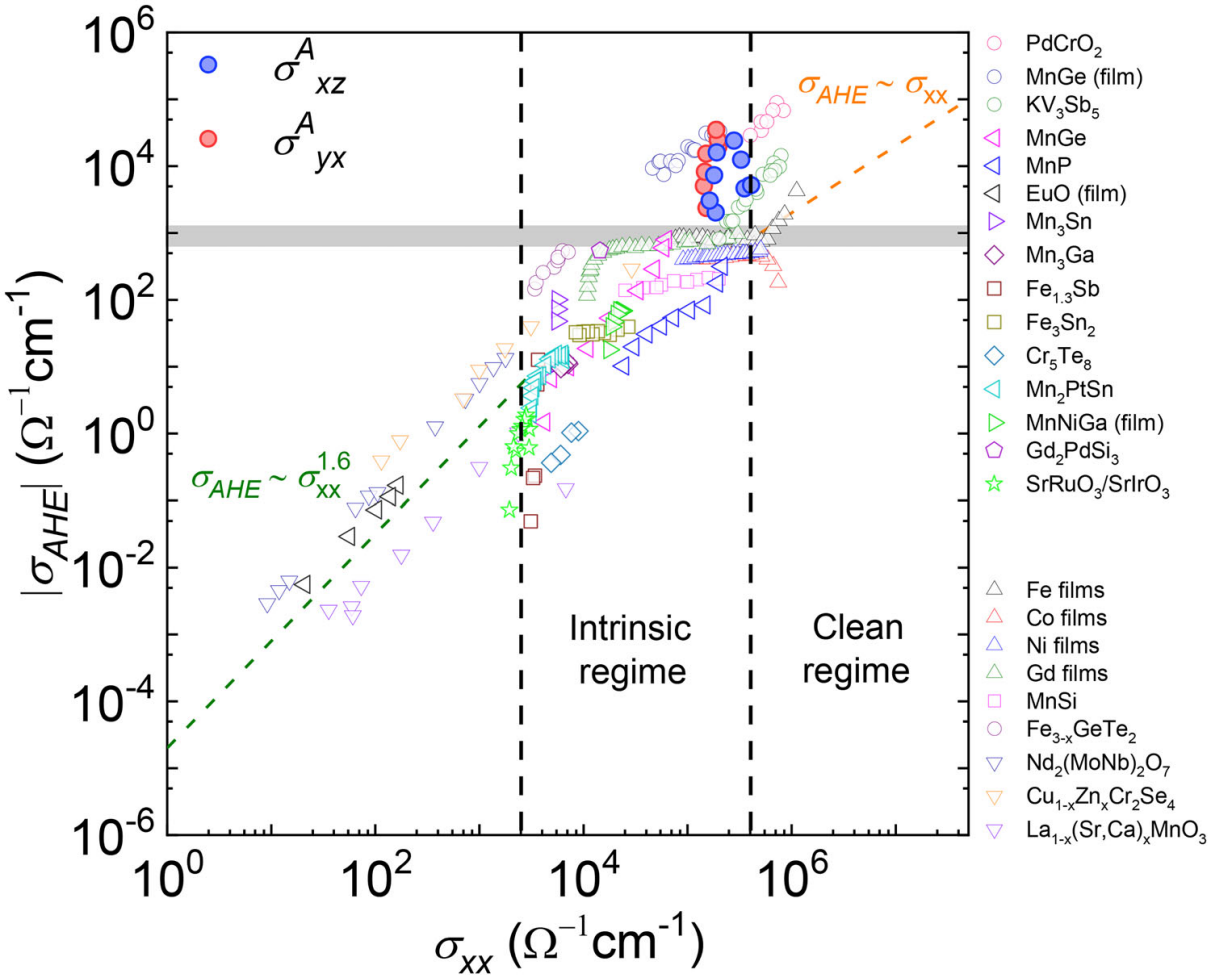
Colossal anomalous Hall conductivity in ${\mathrm{YMn}}_{6}{\mathrm{Sn}}_{6}$. Absolute anomalous Hall conductivity, $|\sigma_{\mathrm{AHE}}|$, plotted as a function of longitudinal conductivity, $\sigma_{xx}$, for ${\mathrm{YMn}}_{6}{\mathrm{Sn}}_{6}$ and representative magnetic materials. The blue and red circles denote the anomalous Hall conductivities of ${\mathrm{YMn}}_{6}{\mathrm{Sn}}_{6}$ measured in two field geometries, $\sigma_{xz}^{A}$ for H∥y and $\sigma_{yx}^{A}$ for H∥z, respectively. The $|\sigma_{\mathrm{AHE}}|$ values for conventional ferromagnetic metals and films, kagome magnets, and frustrated magnetic systems are shown for comparison [3, 40, 41, 47, 49, 60, 61, 62, 63, 64, 65, 66, 67, 68, 69, 70, 71]. The vertical dashed lines separate the dirty, intrinsic, and clean regimes of anomalous Hall transport. The grey horizontal band marks the characteristic intrinsic Berry‐curvature scale, of order $e^{2}/ha∼10^{3}\;\Omega^{-1}{\mathrm{cm}}^{-1}$. Dashed guide lines indicate the empirical scaling behaviors $\sigma_{AHE}\propto \sigma_{xx}^{1.6}$ in the dirty regime and $\sigma_{AHE}\propto \sigma_{xx}$ in the clean regime, where skew scattering is expected to dominate. ${\mathrm{YMn}}_{6}{\mathrm{Sn}}_{6}$ lies close to the clean regime and exhibits an exceptionally large anomalous Hall conductivity, far exceeding the conventional intrinsic scale.

Let us focus on the AHC for H∥y, $\sigma_{xz}^{A}$, in ${\mathrm{YMn}}_{6}{\mathrm{Sn}}_{6}$ at magnetic fields above the magnetization saturation field in the FF phase. As shown in Figure 5, $\sigma_{xz}^{A}$ reaches $∼5.0\times 10^{3}$ S/cm, larger than the highest AHC values reported to date for ferromagnets e.g., $∼1.6\times 10^{3}$ S/cm in Ni [51], $∼2.0\times 10^{3}$ S/cm in ${\mathrm{Co}}_{2}\mathrm{MnGa}$ [52], $∼2.5\times 10^{3}$ S/cm in Fe‐doped ${\mathrm{CoS}}_{2}$ [53], and $∼2.9\times 10^{3}$ S/cm in ${\mathrm{NdCrSb}}_{3}$ [54]. This colossal AHC value far exceeds the intrinsic Berry‐curvature limit $e^{2}/ha\approx 1.0\times 10^{3}$ S/cm [40, 41] and is nearly ∼ 23 times larger than the calculated intrinsic AHC for the FF phase of ${\mathrm{YMn}}_{6}{\mathrm{Sn}}_{6}$. In this FF phase, THE contribution from spin scalar chirality is negligible, because it vanishes with fully aligned spin moments. Instead, this unusually large enhancement can be due to extrinsic mechanisms, most likely impurity skew scattering in the clean limit [40, 41]. With a longitudinal conductivity approaching $∼10^{6}\;S/\mathrm{cm}$, ${\mathrm{YMn}}_{6}{\mathrm{Sn}}_{6}$ indeed lies in a regime where such extrinsic contributions can dominantly contribute to the AHC. Nevertheless, we found that the enhancement factor ∼23 of the measured AHC with respect to the Berry curvature calculation is an order of magnitude larger than in the colossal AHE materials mentioned above, with the typical enhancement ratio of ∼1−4 [51, 52, 53, 54]. Hence, conventional skew scattering driven solely by spin–orbit coupling at impurity sites cannot account for the observed colossal AHC in ${\mathrm{YMn}}_{6}{\mathrm{Sn}}_{6}$.

## 6 Berry Curvature‐Induced Skew Scattering

A more plausible explanation invokes skew‐scattering contributions that are strongly enhanced by Berry curvature, even in the absence of SOC at impurity sites. As shown in Ref. [55], finite Berry curvature induces a noncommutative nature of electron wavefunctions in the real space, producing asymmetric scattering probabilities and thus a significant increase in the AHC. In this framework, the asymmetric scattering rate obeys $W^{\text{asym}}\propto n_{i}V^{3}\;\Omega_{k_{F}}$, where $n_{i}$ is the impurity density, V the scalar impurity potential, and $\Omega_{k_{F}}$ the Berry curvature at the Fermi wavevector. By contrast, in conventional skew scattering driven by SOC at the impurity site, $W^{\text{asym}}\propto n_{i}V^{2}\lambda_{\text{imp}}$, with $\lambda_{\text{imp}}$ the SOC strength at the impurity sites. Consequently, the ratio of the two resulting anomalous Hall conductivities satisfies $\frac{\sigma_{yx}^{\text{BC,sk}}}{\sigma_{yx}^{\text{imp,sk}}}\propto \frac{V\;\Omega_{k_{F}}}{\lambda_{\text{imp}}}$, where $\sigma_{yx}^{\text{BC,sk}}$ and $\sigma_{yx}^{\text{imp,sk}}$ correspond to the AHC contributions from Berry‐curvature‐enhanced skew scattering and impurity skew scattering, respectively. Taking typical values V∼0.1–1 eV, $\Omega_{k_{F}}\;∼\;10^{2}$–$10^{3}\;/{Å}^{2}$, and $\lambda_{\text{imp}}\;∼\;0.05$–1 eV /Å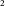, this ratio can reach $10^{1}$–$10^{2}$, indicating that Berry‐curvature‐induced skew scattering is far more effective at amplifying the AHC than conventional impurity‐driven skew scattering. A recent study on colossal AHC of Fe‐ and Ni‐doped ${\mathrm{CoS}}_{2}$ indeed reveals significantly enhanced AHC than the Berry curvature prediction in the clean limit. The doping dependence of this skew‐scattering‐enhanced AHC shows an intimate correlation with the calculated Berry curvature [53], supporting the dominant role of Berry‐curvature‐induced skew scattering. We therefore attribute the remarkably large AHC observed in the FF phase of ${\mathrm{YMn}}_{6}{\mathrm{Sn}}_{6}$ to the combined effect of intrinsic Berry curvature and Berry‐curvature‐enhanced skew scattering in the clean regime.

Below the magnetization saturation field in the FL phases, we observe an even stronger enhancement of $\sigma_{xz}^{A}$, reaching up to $∼2.5\times 10^{4}$ S/cm at H=9.5 T. In the previous studies, the THE has been attributed to scalar spin chirality generated by magnetic fluctuations [21] or nontrivial spin textures [22]. However, the pronounced enhancement of $\sigma_{xz}^{A}$ observed in the FL phase, rather than in the TCS phase, at low temperatures cannot be primarily ascribed to the THE. First, magnetic fluctuations capable of generating scalar spin chirality are expected to be nearly quenched at low temperatures and high magnetic fields, as theoretically confirmed in a recent study [21]. Second, scalar spin chirality should be sizable in the TCS phase owing to its conical spiral spin configuration, whereas it should be strongly suppressed in the FL phase, where the spin configuration has a negligible solid angle in spin space. Consistent with this expectation, recent high‐field neutron diffraction experiments reported scalar spin chiralities of −0.726 at 2 K and 5 T and −0.864 at 240 K and 3 T in the TCS phase [22]. Although these sizable values would suggest a finite THE contribution in the TCS phase, no clear THE signature is observed at low temperatures, in contrast to the pronounced enhancement of $\sigma_{xz}^{A}$ found in the FL phase in our experiments. Therefore, while a minor THE contribution to the AHC cannot be completely ruled out, it is unlikely to be the dominant origin of the colossal $\sigma_{xz}^{A}$ below the saturation field.

The monotonous increase of $\sigma_{yx}^{A}\left(H\right)$ with magnetic field also supports minor contribution of the THE below the saturation field. Under magnetic field applied along the c‐axis, the Mn moments continuously cant from the ab‐plane toward the c direction as shown in Figure 1c, without an additional field‐induced magnetic transition. If the THE contribution due to the finite scalar spin chirality is dominant, $\sigma_{yx}^{A}\left(H\right)$ would become largest at an intermediate noncoplanar spin configuration and then diminish as the moments approach the collinear FF phase. Experimentally, however, $\sigma_{yx}^{A}\left(H\right)$ increases monotonically with magnetic field and remains largest in the FF phase, where the fully aligned moments produce no scalar spin chirality (Figure 4e). This behavior further supports the conclusion that the measured Hall responses are dominated by the AHE rather than by the THE contribution.

The observed colossal AHC below the saturation field can instead be attributed to the pronounced dependence of the Berry curvature on the spin‐canting angle. As shown in Figure 4f, the calculated Berry‐curvature contribution to $\sigma_{xz}^{A}$ exhibits a maximum near H∼9.5 T, consistent with the field at which the experimental $\sigma_{xz}^{A}$ reaches its peak. This field‐tunable Berry curvature can strongly enhance $\sigma_{xz}^{A}$ through the skew‐scattering channel, as discussed above for the FF phase. By assuming the same enhancement factor of ∼23 obtained for the FF phase, the renormalized $\sigma_{xz}^{A}$ reproduces the experimentally observed peak structure in Figure 4f. Although this analysis relies on an approximate double‐AFM spin configuration and a field‐independent enhancement factor, the qualitative agreement between the experimental and calculated $\sigma_{xz}^{A}$ highlights the important role of spin‐canting‐angle‐dependent Berry curvature. These observations suggest that the complex field dependence and the colossal enhancement originate from the synergistic effects of strongly field‐tunable k‐space Berry curvature and Berry‐curvature‐driven skew scattering as the system approaches the clean limit.

The resulting AHC in ${\mathrm{YMn}}_{6}{\mathrm{Sn}}_{6}$ reaches $∼2.5\times 10^{4}$ S/cm, among the highest values reported for magnetic materials. Figure 5 compares the absolute AHC, $|\sigma_{\mathrm{AHE}}|$, with the longitudinal conductivity, $\sigma_{xx}$, for ${\mathrm{YMn}}_{6}{\mathrm{Sn}}_{6}$ and representative ferromagnetic, kagome, and frustrated magnetic systems [3, 40, 41, 47, 49, 60, 61, 62, 63, 64, 65, 66, 67, 68, 69, 70, 71]. For both Hall components of ${\mathrm{YMn}}_{6}{\mathrm{Sn}}_{6}$, $\sigma_{xz}^{A}$ for H∥y and $\sigma_{yx}^{A}$ for H∥z, the AHC lies well above the conventional intrinsic Berry‐curvature scale of $∼10^{3}$ S/cm, while the corresponding longitudinal conductivities place ${\mathrm{YMn}}_{6}{\mathrm{Sn}}_{6}$ close to the clean transport regime, where skew scattering is expected to scale approximately linearly with $\sigma_{xx}$. Moreover, the maximum $|\sigma_{\mathrm{AHE}}|$ values of ${\mathrm{YMn}}_{6}{\mathrm{Sn}}_{6}$ exceed most reported values for frustrated magnets, with the notable exception of the recent record value reported for the ultraclean frustrated antiferromagnet ${\mathrm{PdCrO}}_{2}$ [71]. These observations indicate that the observed AHC cannot be accounted for by the intrinsic Berry‐curvature contribution or by a real‐space topological Hall contribution alone, and instead point to a substantial extrinsic amplification of AHC near the clean regime. We note that the colossal AHC in ${\mathrm{PdCrO}}_{2}$ has been attributed to spin‐cluster skew scattering by fluctuating clusters of localized spins with finite scalar spin chirality, a contribution that is strongly amplified in the clean limit [71]. Together with our findings in ${\mathrm{YMn}}_{6}{\mathrm{Sn}}_{6}$, this comparison suggests that clean‐limit skew scattering, despite its distinct microscopic origins, provides a general route to enhancing the AHC.

In conclusion, we demonstrate that, in kagome antiferromagnets, field‐induced changes in spin configuration, including spin canting, can substantially modify the low‐energy electronic band structure and generate strongly field‐dependent Berry curvature in momentum space. This finding challenges the commonly adopted assumption that the AHC arising from momentum‐space Berry curvature should scale proportionally with the field‐dependent magnetization [19, 35, 36, 45, 46, 47, 48, 49]. Within this conventional framework, any additional contribution deviating from the $\sigma^{A}\left(H\right)\propto M\left(H\right)$ behavior is often attributed to a THE driven by field‐induced scalar spin chirality in real space. However, kagome antiferromagnets host flat bands and Dirac band crossings near the Fermi level, which act as strong sources of k‐space Berry curvature. Even subtle modifications of these electronic structures caused by spin canting can therefore produce a complex field dependence of the AHC. This effect is further enhanced as the system approaches the clean limit, where Berry‐curvature‐induced skew scattering can amplify the contribution from k‐space Berry curvature. The colossal AHC and its strong magnetic‐field tunability in ${\mathrm{YMn}}_{6}{\mathrm{Sn}}_{6}$ thus reveal a complex interplay between magnetic configuration and momentum‐space Berry curvature in kagome antiferromagnets.

## 7 Methods

### 7.1 Single Crystal Growth and Physical Property Measurements

Single crystals of ${\mathrm{YMn}}_{6}{\mathrm{Sn}}_{6}$ were grown using a Sn‐flux method with a Canfield crucible. The stoichiometric mixture (1:6:30) of Y chips (99.99%), Mn powder (99.99%), and Sn shot (99.99%) was loaded in a sealed quartz tube under vacuum and loaded into a box furnace at 1000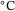 for 24 h, cooled down to 600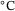 and centrifuged. Flat and shiny crystals were obtained, of which crystallinity and stoichiometry were confirmed by X‐ray diffraction and energy‐dispersive X‐ray spectroscopy (Figure 1). Magnetization was measured under a magnetic field along the c‐axis or ab‐plane using a vibrating sample magnetometer of Physical Property Measurement System (PPMS‐14T, Quantum Design). The conventional six‐probe method was employed for the resistivity measurements. The samples were measured in a Physical Property Measurement System (Quantum Design). We attached gold wires on a rectangular‐shaped sample and applied the current (I) along a‐axis or c‐axis and measured voltages ($V_{mea}$) in longitudinal and transverse directions while sweeping magnetic fields (H) from 14 T to ‐14 T. The longitudinal ($R_{xx,zz}\left(H\right)$) and transverse ($R_{yx,xz}\left(H\right)$) resistances are extracted from the data taken with positive and negative magnetic fields, using $R_{xx,zz}\left(H\right)=\left[V_{mea}\left(H\right)+V_{mea}\left(-H\right)\right]/2I$ and $R_{yx,xz}\left(H\right)=\left[V_{mea}\left(H\right)-V_{mea}\left(-H\right)\right]/2I$.

### 7.2 Angle‐Resolved Photoemission Spectroscopy

ARPES measurements were conducted at the 4A2 beamline of the Pohang Light Source using a DA30L electron analyzer (Scienta Omicron, Sweden) and at the 4.0.3 beamline of Advanced Light Source. Crystal was cleaved under ultrahigh vacuum (base pressure < 1 × $10^{-10}$ Torr) to obtain clean surfaces. At the Pohang Light Source, both cleaving and measurements were performed at 70 K, whereas at the Advanced Light Source, cleaving and measurements were carried out at 10 K. Furthermore, measurements with a photon energy of 138 eV and both linear‐horizontal (LH) and linear‐vertical (LV) polarizations were performed on the kagome termination to analyze orbital characters in detail.

### 7.3 Density Functional Theory Calculations

We performed first‐principles calculations within the framework of DFT using the Perdew–Burke–Ernzerhof‐type generalized gradient approximation for the exchange‐correlation functional, as implemented in the Vienna ab initio simulation package [56, 57]. All calculations were carried out using the kinetic energy cutoff of 520 eV on a 12 × 12 × 4 Monkhorst–Pack k‐point mesh for the double‐layer AFM spin configuration of ${\mathrm{YMn}}_{6}{\mathrm{Sn}}_{6}$. The intrinsic anomalous Hall conductivity is calculated based on the Wannier90 code [58, 59] with d orbitals of Mn atoms and p orbital of Sn atoms. The k mesh for intrinsic Hall conductivity calculation is 130 × 130 × 30 with adaptive refinement mesh of 5 × 5 × 5.

## Author Contributions

J.S.K., M.C., H.K. conceived the experiments. M. C. grew high‐quality single crystals. M.C., H.K., H.S., J.C., Y.J. and J.S.K. performed the magnetotransport measurements at high magnetic fields. K.H.J. performed DFT calculations, and M.S., J.Y.K. and H.W.Y. carried out angle‐resolved photoemission spectroscopy measurements and analysis. M.C., H.K., M.S., H.W.Y., K.H.J., and J.S.K. co‐wrote the manuscript. All authors discussed the results and commented on the paper.

## Conflicts of Interest

The authors declare no conflicts of interest.

## Supporting information


**Supporting File**: advs77738‐sup‐0001‐SuppMat.pdf.

## Data Availability

The data that support the findings of this study are available from the corresponding author upon reasonable request.
