# Supplementary material for: Colossal Magnetic‐Field‐Induced Anomalous Hall Conductivity in a Kagome Antiferromagnet YMn6Sn6
Source: Adv Sci (Weinh). 2026 Sep 21:e77738. Online ahead of print. doi: 10.1002/advs.77738 (PMC13591356; doi:10.1002/advs.77738)
Supplement: Supplementary file 1 [file ADVS-9999-e77738-s001.pdf]

# Supplementary Materials: Colossal magnetic-field-induced anomalous Hall conductivity in a kagome antiferromagnet $YMn_6Sn_6$


Minhyuk Choi,[1,2,*] Hoil Kim,[3,*] Minki Sung,[1,2,*] Hyeong Woo Seo,[1,2] Jun Young Choi,[4] Jaeyoung Kim,[1] Youn Jung Jo,[4] Han Woong Yeom,[1,2,†] Kyung-Hwan Jin,[5,‡] and Jun Sung Kim[1,2,§]

[1] *Department of Physics, Pohang University of Science and Technology, Pohang 37673, Republic of Korea*

[2] *Center for Artificial Low Dimensional Electronic Systems, Institute for Basic Science (IBS), Pohang 37673, Republic of Korea*

[3] *Institute for Materials Research, Tohoku University, Sendai 980-8577, Japan*

[4] *Department of Physics, Kyungpook National University, Daegu 41566, Republic of Korea*

[5] *Department of Physics and Research Institute of Materials and Energy Sciences, Jeonbuk National University, Jeonju 54896, Republic of Korea*


---


[*] equal contribution

[†] yeom@postech.ac.kr

[‡] khwanjin@jbnu.ac.kr

[§] js.kim@postech.ac.kr

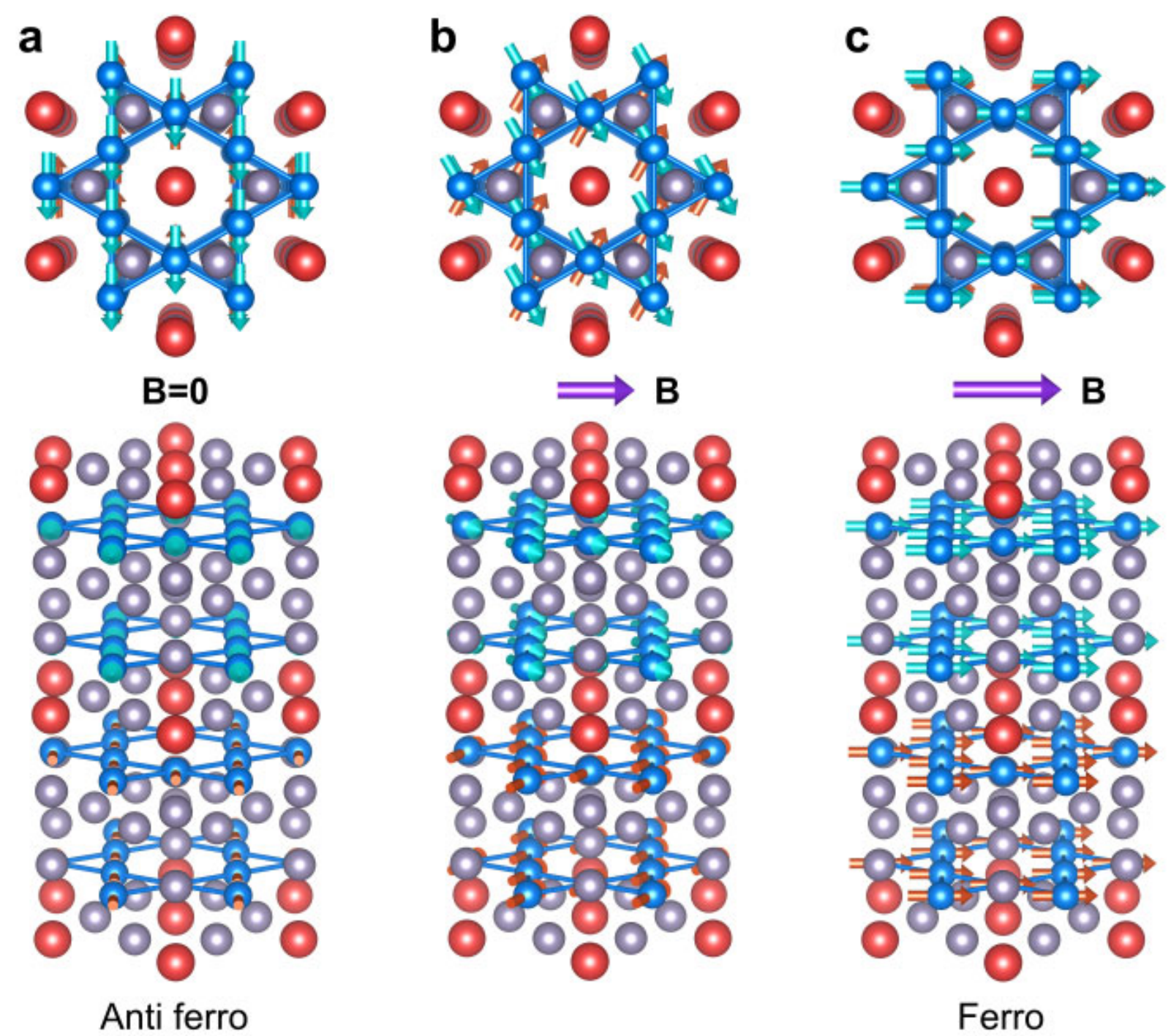


Supplementary Fig. 1. **Top and side views of the double-layer antiferromagnetic configuration under an in-plane magnetic field.** Schematic illustration of Mn spin arrangements in $YMn_6Sn_6$ for **a**, zero field ($\theta = 0°$, antiferromagnetic), **b**, intermediate canting angle $\theta$ under a finite in-plane magnetic field, and **c**, fully aligned ferromagnetic state ($\theta = 90°$). The canting angle $\theta$ denotes the deviation of Mn spins from the zero-field AFM orientation toward the applied field direction. Increasing $\theta$ corresponds to a continuous evolution from the AFM configuration to the ferromagnetic ordering.

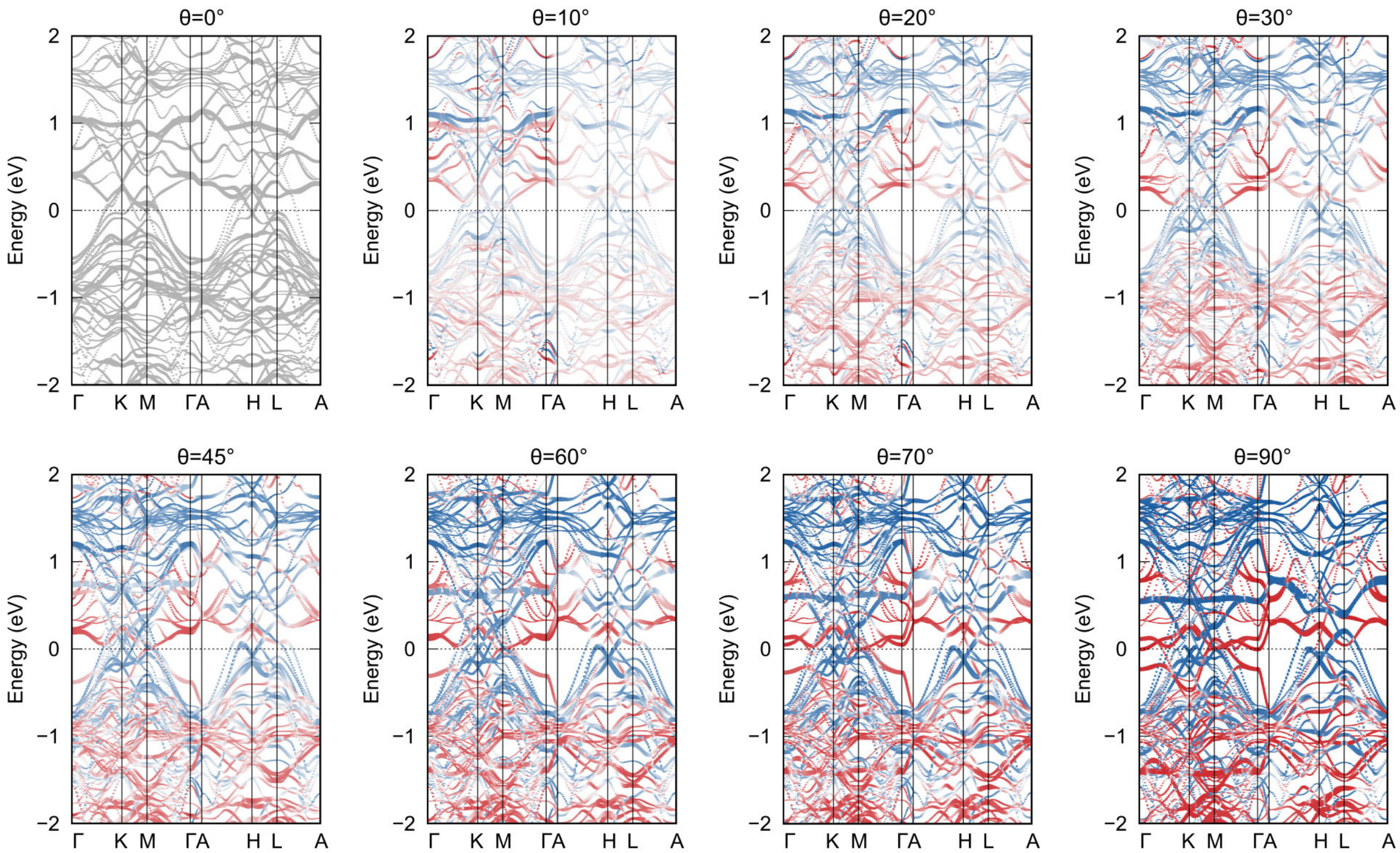


Supplementary Fig. 2. **Spin-canting-angle dependence of Mn $d_{z^2}$-projected band structures under spin canting.** Calculated band structures for canting angle $\theta = 0°$ to $90°$, projected onto the Mn $d_{z^2}$ orbital, with dot size proportional to the orbital weight. The red-blue color gradient denotes spin polarization along the applied in-plane magnetic field direction. As $\theta$ increases from the antiferromagnetic (0°) toward the ferromagnetic (90°) configuration, kagome-derived flat bands exhibit pronounced spin splitting and shift toward the Fermi level, accompanied by enhanced $k_z$ dispersion.

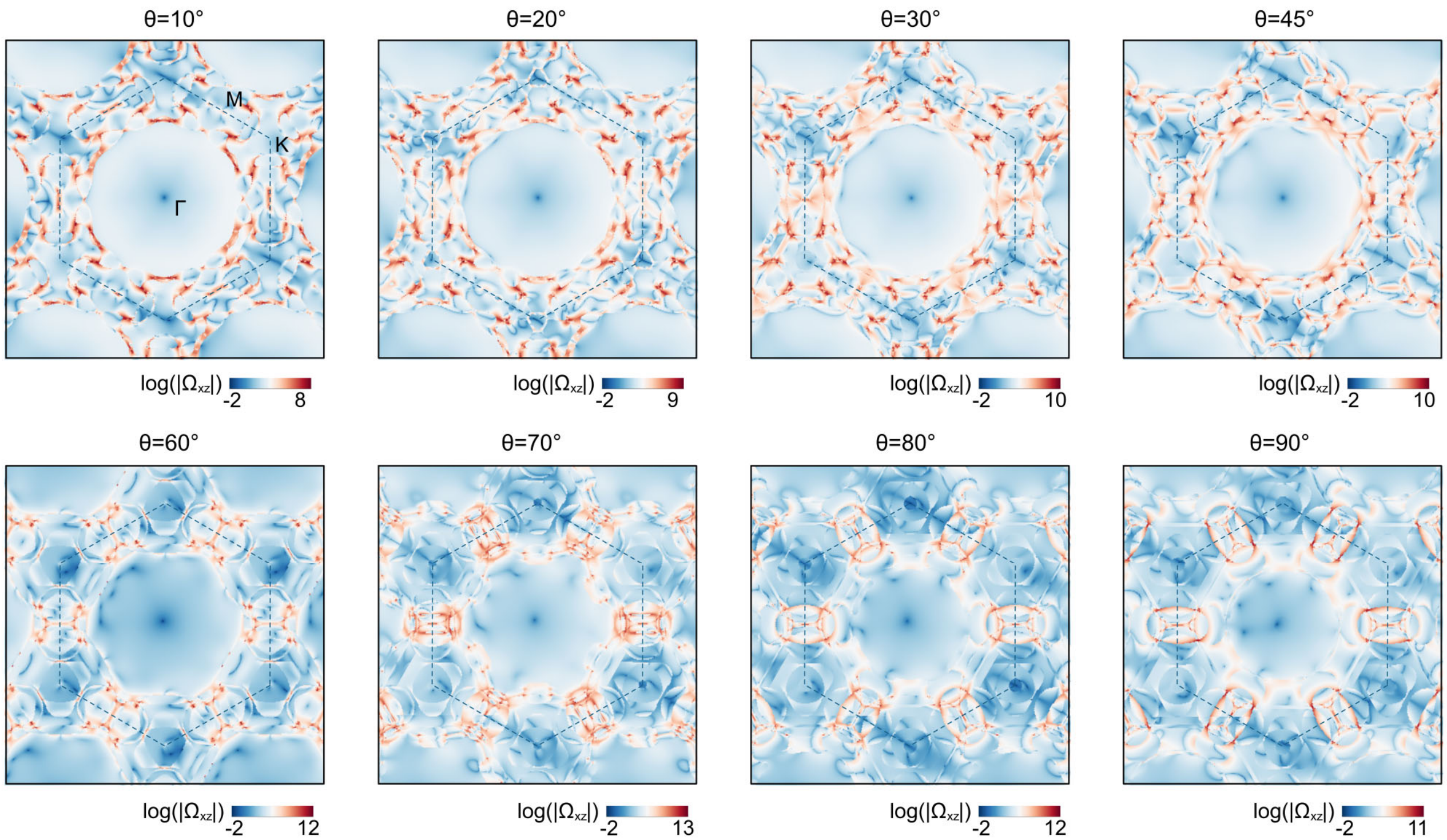


Supplementary Fig. 3. **Evolution of Berry curvature distribution with spin canting angle.** Logarithmic scale maps of the Berry curvature magnitude $|\Omega_{xz}|$ in the $k_z = 0$ plane for canting angle $\theta = 10^\circ$ to $90^\circ$. The canting angle $\theta$ is defined as the rotation of Mn spins from the zero-field antiferromagnetic configuration toward the in-plane magnetic field direction. As $\theta$ increases, the Berry curvature distribution undergoes significant changes, with pronounced hotspots emerging near the M points and reaching maximum intensity around $\theta \approx 70^\circ$, consistent with the calculated peak in quantum anomalous Hall conductivity.

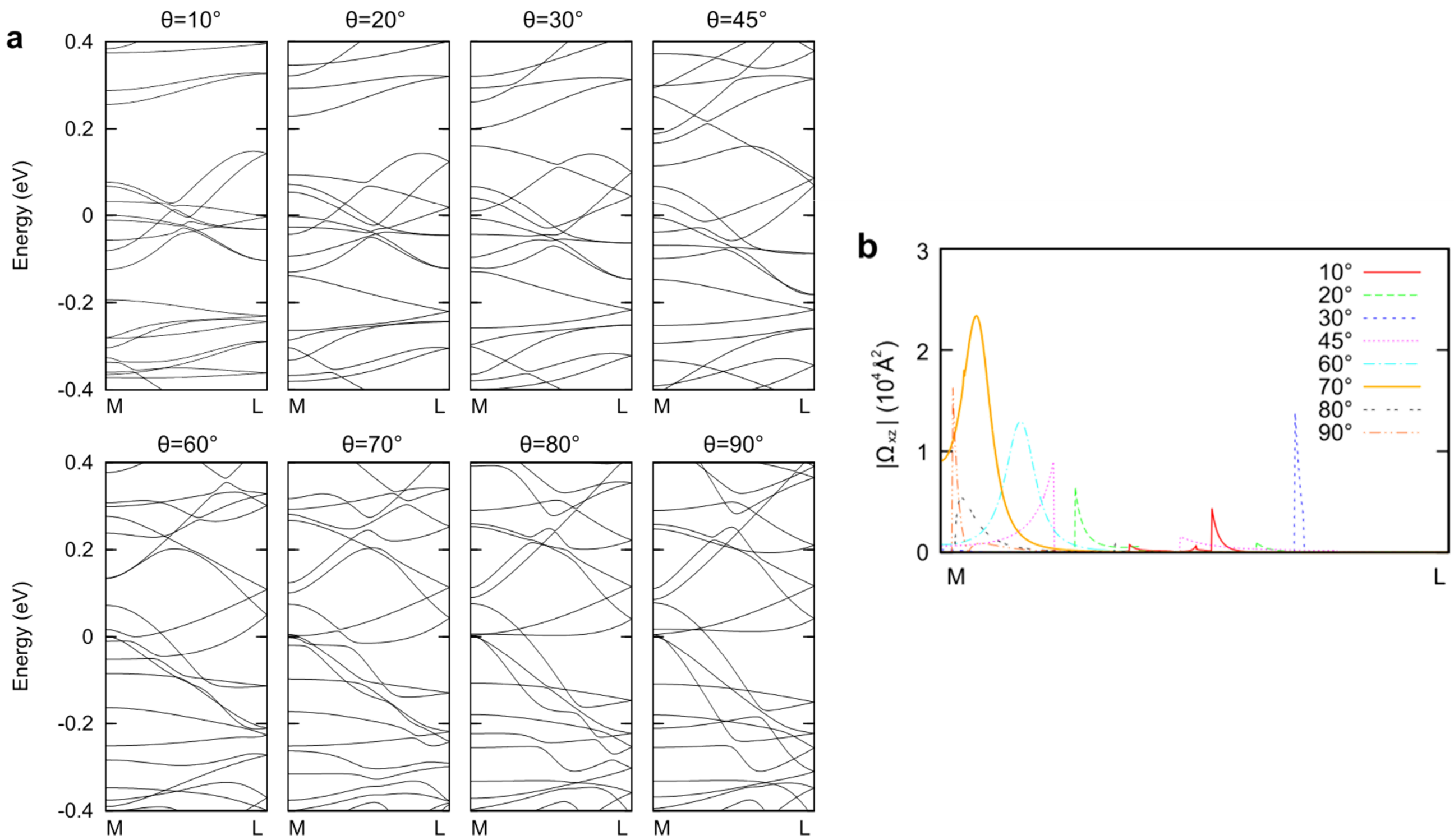


Supplementary Fig. 4. **Spin-canting-angle dependence of band structures and Berry curvature along the M-L path.** **a**, Calculated band structures along the M-L direction for canting angles $\theta = 10° - 90°$. The evolution of the spin-canted state modifies the band dispersion, with a pronounced SOC-induced gap emerging near the M point for $\theta \approx 70°$. **b**, In-plane Berry curvature magnitude $|\Omega_{xz}|$ along the M-L path for the corresponding canting angles. A sharp enhancement near the M point is observed at $\theta \approx 70°$, correlating with the gap opening in panel **a** and the peak in the calculated quantum anomalous Hall conductivity.

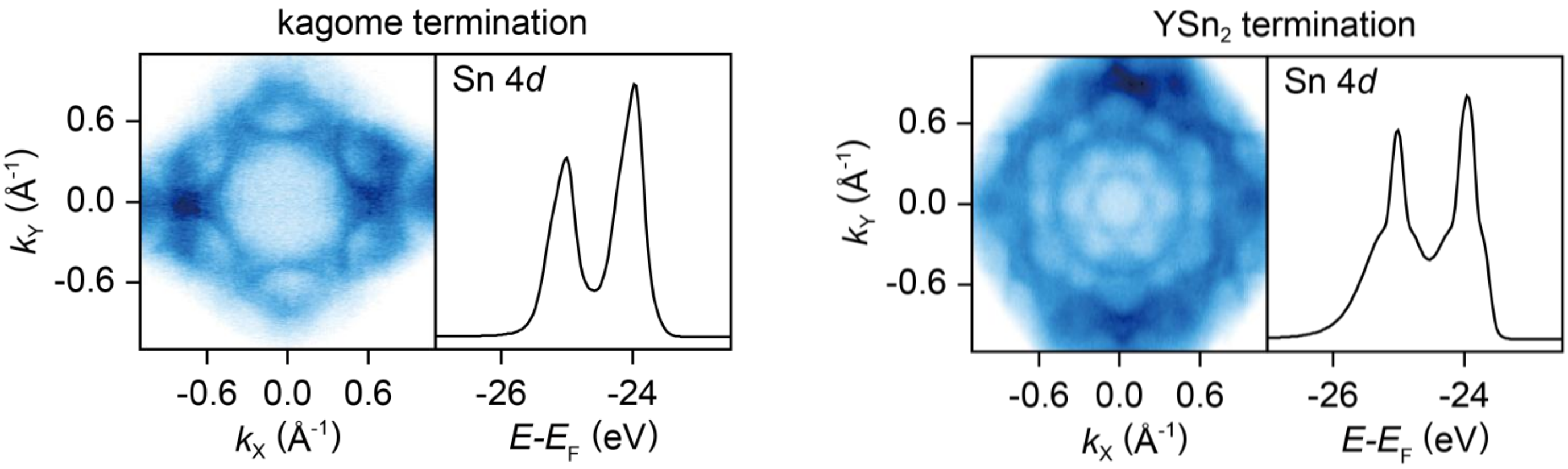


Supplementary Fig. 5. **Distinct electronic structures of kagome- and $YSn_2$-terminated surface.** ARPES Fermi surface maps and corresponding Sn 4*d* core-level spectra measured on the kagome-terminated (left) and $YSn_2$-terminated (right) surfaces. Distinct Fermi surface features are observed depending on the surface termination.

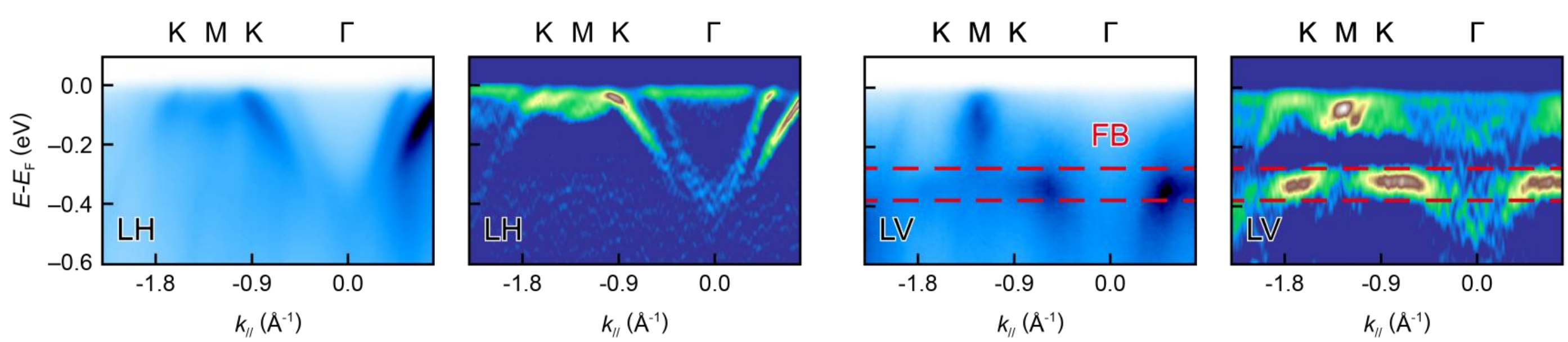


Supplementary Fig. 6. **Observation of flat band in polarization-dependent ARPES along ΓK direction.** Polarization-dependent ARPES spectra measured along the ΓK direction using LH and LV polarizations. The flat band at −0.4 eV is more clearly resolved in the second derivative plots.

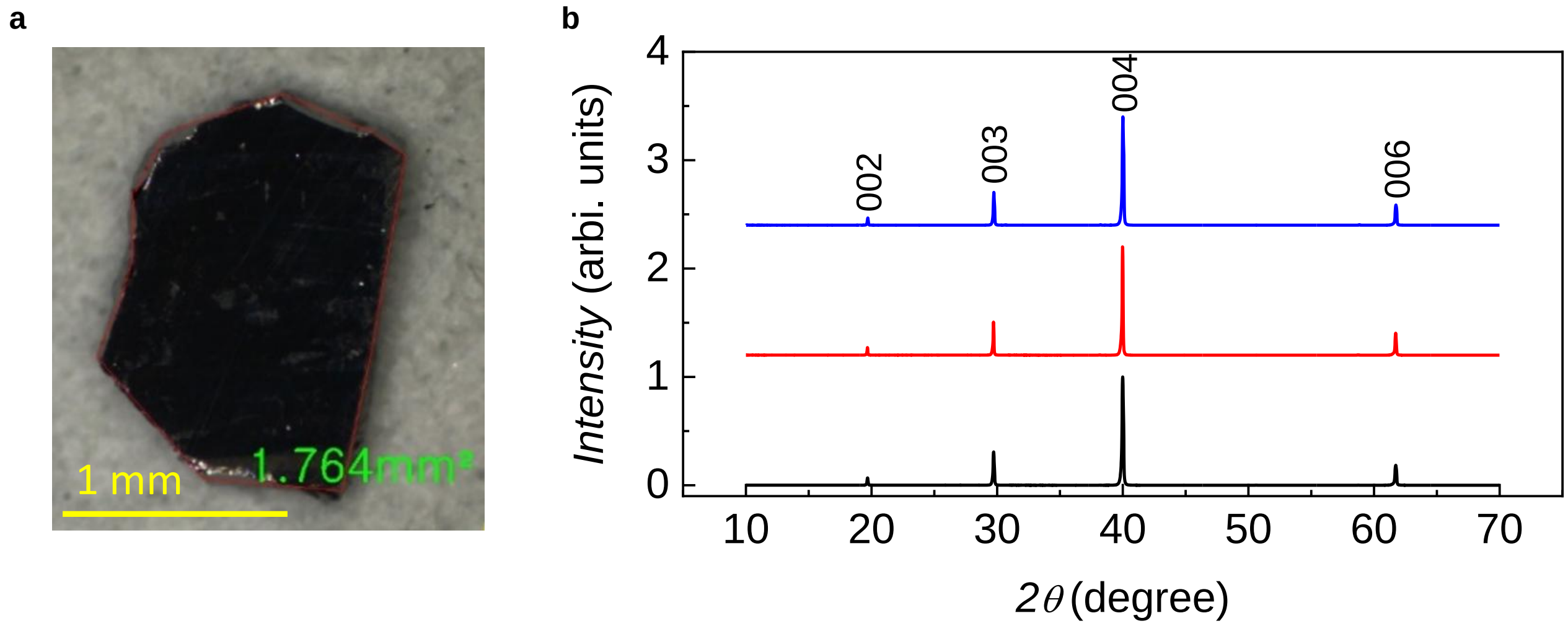


Supplementary Fig. 7. **Single crystal of $YMn_6Sn_6$.** **a**, Optical microscope image of a representative single crystal exhibiting a well-defined hexagonal plate-like morphology with a smooth surface (area $\sim 1.76$ mm$^2$). **b**, Out-of-plane X-ray diffraction patterns measured from several single crystals. (00l) reflections are observed, confirming that the broad facets correspond to the *ab*-plane and indicating high crystallographic orientation among the measured crystals.

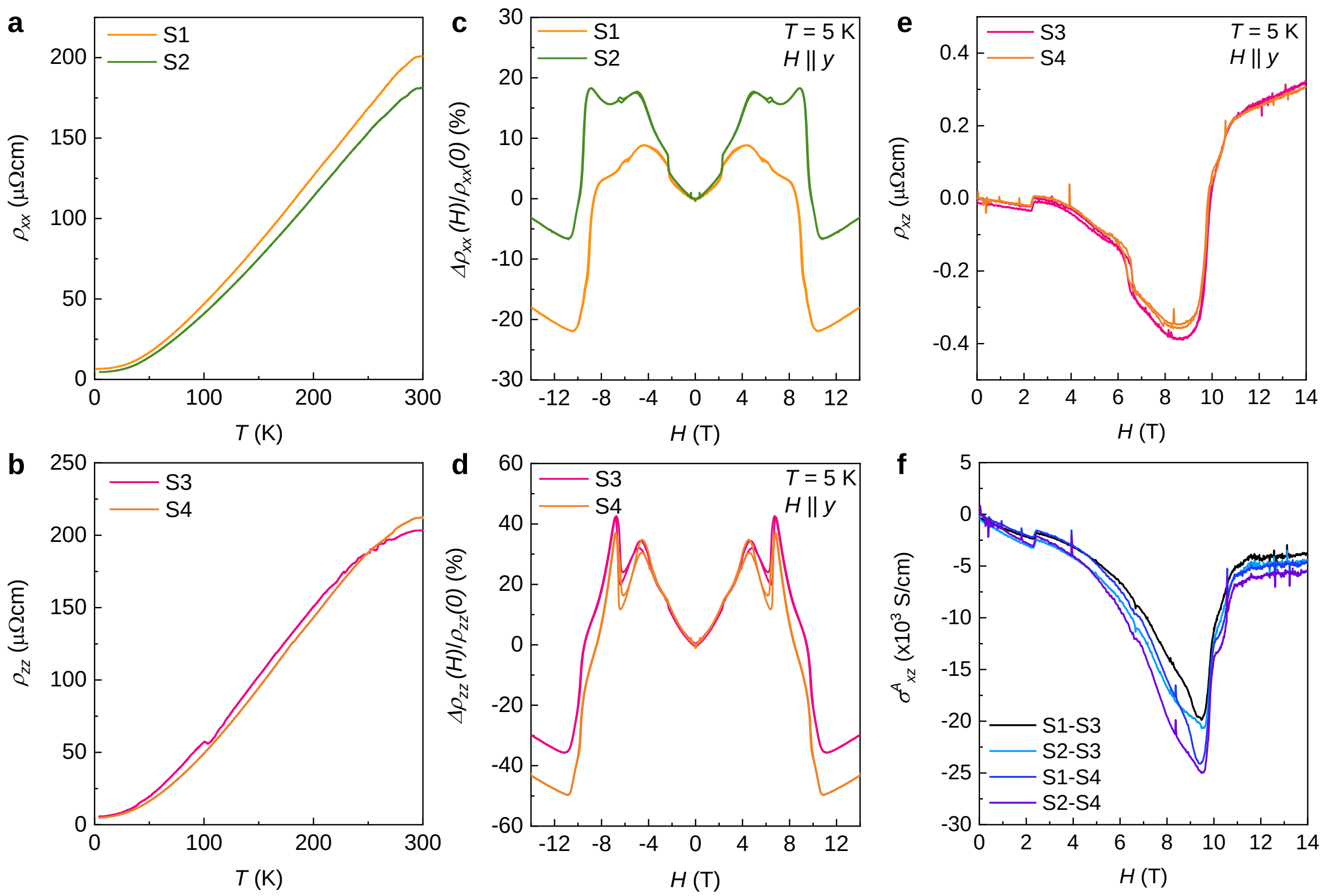


Supplementary Fig. 8. **Temperature- and field-dependent transport properties of different single crystals.** **a**, **b**, Temperature dependence of the longitudinal resistivity, $\rho_{xx}$ for samples S1−S2 and $\rho_{zz}$ for samples S3−S4, respectively, showing clear metallic behavior. **c**, **d**, Magnetic-field dependence of the normalized magnetoresistance $\Delta\rho_{xx}(H)/\rho_{xx}(0)$ and $\Delta\rho_{zz}(H)/\rho_{zz}(0)$ at 5 K for $H \parallel y$, exhibiting pronounced kinks that are consistent across different crystals. **e**, Field dependence of the Hall resistivity $\rho_{xz}$ for S3 and S4 at 5 K, showing a clear anomalous Hall contribution near 9 T. **f**, Corresponding anomalous Hall conductivity $\sigma^A_{zx}$ calculated from multiple sample pairs, confirming the reproducibility of the transport features among crystals.
